# Non-Equilibrium Potential Responses towards Neutral Orcinol Using All-Solid-State Potentiometric Sensors Integrated with Molecularly Imprinted Polymers

**DOI:** 10.3390/polym11081232

**Published:** 2019-07-25

**Authors:** Saad S. M. Hassan, Abd El-Galil E. Amr, Nada H. A. Elbehery, Mohamed A. Al-Omar, Ayman H. Kamel

**Affiliations:** 1 Chemistry Department, Faculty of Science, Ain Shams University, 11566 Abbasia, Cairo, Egypt; 2 Pharmaceutical Chemistry Department, Drug Exploration & Development Chair (DEDC), College of Pharmacy, King Saud University, Riyadh 11451, Saudi Arabia; 3 Applied Organic Chemistry Department, National Research Centre, 12622 Dokki, Giza, Egypt

**Keywords:** sensors, solid-contact ISEs, molecularly imprinted polymers (MIPs), orcinol, neutral response mechanism

## Abstract

Molecularly imprinted polymer (MIP) receptors have been synthesized, characterized, and applied as new selective receptors in solid-contact ion selective electrodes (ISEs) towards non-dissociated 3,5-dihydroxytoluene (orcinol). Two monomers, namely methacrylic acid (MAA) and acrylamide (AA), were used in the preparation of MIP receptors. Graphene (Gr) was used as the solid contact material between the sensing membrane and the electrical contact substrate. Based on non-equilibrium sensing mechanism, the proposed sensors reveal observably enhanced detection sensitivity towards orcinol with detection limits 1.7 × 10^−5^ and 3.3 × 10^−6^ M for sensors based on MIP/MAA and MIP/AA, respectively. The selectivity coefficients measured by the modified separate solution method (MSSM) for the proposed sensors showed good selectivity towards orcinol over most common other phenols and inorganic anions. All measurements were made in the presence of 30 mM phosphate buffer solution (PBS) with a pH of 7.0. Potential stability for the proposed sensors was tested by constant-current chronopotentiometry. No water films were formed between the sensing membrane and the electron conductor substrate. The applicability of MIP/MAA incorporated ISE has been checked by recovery test of orcinol in the presence of soil matrix and by standard addition method.

## 1. Introduction

In recent years, potentiometric sensors, or so-called “polymeric membrane ion-selective electrodes (ISEs)”, have become an attractive tool for determination of ionic species in different fields, such as clinical diagnostics and trace-level environmental assessment [1,2,3,4,5]. This can be attributed to their intrinsic advantages, such as high selectivity, ease of use, and good reliability. Different ISEs were reported in the literature by our group for quantifying different organic species in different fields, including drugs [6,7], pesticides [8,9], organic solvents [10,11], and biomedical species [4,5,12]. So far, producing new potentiometric sensors for organic ions is a good trend for these types of devices in the field of analytical chemistry. 

Molecularly-imprinted polymers (MIPs) are synthetic receptors for selective recognition of a wide range of analytes with high affinities and selectivity. Integration of molecularly imprinted polymers (MIPs) with potentiometric sensors is now exhibiting a great potential to significantly change the view of using non-affordable ionophores, which are characterized by their high cost, or using ion exchangers, which are characterized by their poor selectivity. On the other hand, MIPs are thermally stable, costly effective, and easier to synthesize [13]. Currently, potentiometric sensors based on different MIP receptors have been synthesized for determination of different organic species in either their ionic or neutral forms [14,15,16]. In spite of their success in the determination of organic species, almost all of these previously reported ISEs integrated with MIPs are in classical liquid-contact mode. This sensor design is characterized by its lower detection limit because of zero-current trans-membrane ion fluxes [17], but has serious limits for their use in environmental analysis. Some of the drawbacks of this design are: (i) the inner solution is exposed to evaporation and is sensitive to changes in the temperature and pressure of the measured sample; (ii) an osmotic pressure can be produced due to the differences in the ionic strength of the sample and the inner filling solution. This pressure produces a net liquid transport from or to the inner filling solution, which may result in volume changes and may provoke delamination of the ion sensing membrane (ISM) [18]; (iii) the implementation of this kind of ISE in flow systems also has the handicap of pressure issues, and moreover, the ISM is affected by back pressure when the electrodes take measurements at depth in water [19].

Solid-contact ISEs eliminate the inner filling solution and have been identified as the new ISE generation [20,21]. This design has different features, such as ease of storage and maintenance, in addition to miniaturization [22]. This design also can offer a lower detection limit because of diminished ion fluxes [23]. The presence of the “blocked” interface between the electronic conductor and ion-selective membrane (ISM) is removed by the insertion of solid-contact materials, such as carbon nano-structures, conducting polymers, or nano-noble metals. Signal noises and potential drifts, which can restrict the applications of ISEs, are now removed [24].

Orcinol is an organic compound that occurs in many species of lichens [25], including Roccella tinctoria and Lecanora. Orcinol can be used in the “toxic glue” of the ant species Camponotus saundersi. It is also used in the production of the dye orcein and as a reagent in some chemical tests for pentoses and hexoses [26] and RNA [27]. 

In this work, novel MIP-based potentiometric sensors were presented for selective and sensitive detection of 3,5-dihydroxy toluene (orcinol) in its neutral form. The sensors were prepared using glassy carbon substrate (GC) in the presence of graphene (Gr) as a solid contact material between the sensing membrane and GC substrate. For comparison, liquid-contact ISEs were also prepared and characterized, then compared with the solid-contact ISEs. The unexpected potential responses to electrically neutral orcinol of polymeric membranes doped with MIP particles as a selective receptor for the recognition of orcinol, along with lipophilic anion exchangers, are presented. It is shown that the ISEs based on MIP particles could produce a high sensitivity towards orcinol and also provides good selectivity over many mono-, di-, and trihdroxyphenols.

## 2. Materials and Methods

### 2.1. Apparatus

All potential measurements were carried out at ambient temperature using an Orion 720/SA pH/mV meter (Cambridge, MA, USA). All spectrophotometric measurements were carried out using a Thermo scientific Evolution 300 spectrophotometer (Waltham, MA, USA). Infrared spectra were carried out by Thermo scientific Nicolet 6700 (Waltham, MA, USA) Chronopotentiometric measurements were performed by applying a constant currents of ±1 nA for 60 s on the screen-printed sensors in presence and absence of graphene (Gr) by using an Autolab Model 2000 potentiostat/galvanostat (Metrohom Instruments, Herisau, Switzerland). A three electrode system, including the ISE working electrode, Ag/AgCl (3 M) as the reference, and a Pt wire as the counter electrode, was used for chronopotentiometric measurements.

For hydrodynamic analysis, an Ismatech peristaltic pump (MS-REGLO, Wertheim, Germany) was used, provided with two channels and a 4-port injection valve (Omnifit, Cambridge, UK) connected with a sample loop volume of 100 μL. The signals were recorded using data acquisition (eight-channel electrode-computer interface, Nico-2000 Ltd., London, UK) set by Nico-2000 software).

### 2.2. Reagents and Materials

All aqueous solutions were prepared using freshly deionized water with specific resistance of 18.2 MΩ cm. The 3,5-Dihydroxytoluene (Orcinol) was obtained from Merck Sharp and Dohme Co. (USA). Ethylene glycol dimethacrylate (EGDMA), acrylamide (AA), methacrylic acid (MAA), high molecular weight poly(vinyl chloride) (PVC), tridodecylemethyl ammonium chloride (TDMAC), graphene (Gr), dioctylphthalate (DOP), and tetrahydrofuran (THF) were used as received from Fluka. Benzoyl peroxide (BPO), ethanol, phenol, 4-methyl catechol, resorcinol, 3,4-dihydroxy toluene (3,4-DHT), and acetic acid were purchased from Sigma Chemicals Co. (St. Louis, MO, USA). A stock solution of orcinol was prepared by dissolving the solid material in 30 mM phosphate buffer solution (PBS), pH 7, and then diluted to various concentrations of working solutions with the same buffer solution. 

### 2.3. Polymer Synthesis and Characterization

Synthesis of orcinol-imprinted polymers was carried out via transferring 0.5 mmol of the orcinol template, 4 mmol of the functional monomer (either MAA or AA), 10 mmol of the cross linker (EGDMA), and 50 mg BPO as initiators to a glass-capped bottle. The components were dissolved in 15 mL acetonitrile as a porogenic solvent. The solution was sonicated, de-gassed with N_2_ gas for 10 min, and then polymerized at 60 °C for 18 h in an oil bath. The obtained polymer particles were refluxed for 20 h with a mixture of ethanol: acetic acid (4:1 *v*/*v*) to remove any un-reacted species and the template. The polymers were left to dry at room temperature to be used in further potentiometric analysis. The non-imprinted polymers (NIPs) were prepared in the same way but without adding the template. For adsorption capacity evaluation, the binding experiments for MIPs were made by immersing 20 mg of the washed polymer in 10 mL of different concentrations of orcinol ranging from 1.2 to 2.8 mM. The mixtures were stirred continuously for 12 h at room temperature. The amount of orcinol in the supernatant after polymer separation was measured spectrophotometrically at 274 nm.

### 2.4. Membranes and Electrodes Construction

The PVC polymeric membranes were prepared by dissolving 550 mg of membrane components in a 4 mL THF into a glass ring (50 mm i.d.) fitted on a glass plate. The solvent was left to evaporate overnight. The formed membranes were peeled off and 4-mm-diameter membrane discs were cut from the membrane and glued to a PVC tube with a THF/PVC slurry. The MIP (NIP) membrane contained 30 mg MIP (NIP), 5 mg TDMAC, 350 mg DOP, and 190 mg PVC. This electrode body was filled with equal volumes of 0.01 M of orcinol and 0.01 M NaCl. 

For all-solid-state orcinol-selective electrodes, 10 µL of graphene solution (0.5 mg in 1 mL THF) was applied by drop casting onto the surface of the glassy carbon substrate (GC) and was left to dry at ambient temperature. After modification, 100 µL of the membrane cocktail was drop casted on the GC/graphene electrode. After being dried for 4 h at room temperature, the electrodes were conditioned in 10 mM orcinol for 24 h before measurement and stored in the same solutions when they were not being used. For comparison, the GC electrode without modification was prepared by putting the above-mentioned membrane directly on the glassy carbon substrate.

For flow injection measurements, the carrier solution was 30 mM PBS (pH 7) with a flow rate 3.5 mL/min. The 100 µL aliquots of series of orcinol concentrations were injected into the carrier stream. The peak height average for three replicate runs was measured.

## 3. Results and Discussions

### 3.1. Synthesis of MIPs and Their Characterization

Molecular imprinting technique is of a great interest, which is used in tailoring a specific recognition site in a polymeric backbone for many different molecular or ionic species. Based on this technique, orcinol-imprinted polymers could be synthesized using non-covalent imprinting protocol. Methacrylic acid (MAA) or acrylamide (AA) were chosen for the preparation of the MIP particles using the ethylene glycol dimethacrylate (EGDMA) as the cross linker. The two used monomers were bound to the template through hydrogen bond formation between either carboxylic −COOH groups in MAA or amide group −NH_2_ in AA with the phenolic –OH in orcinol.

**Figure 1:**
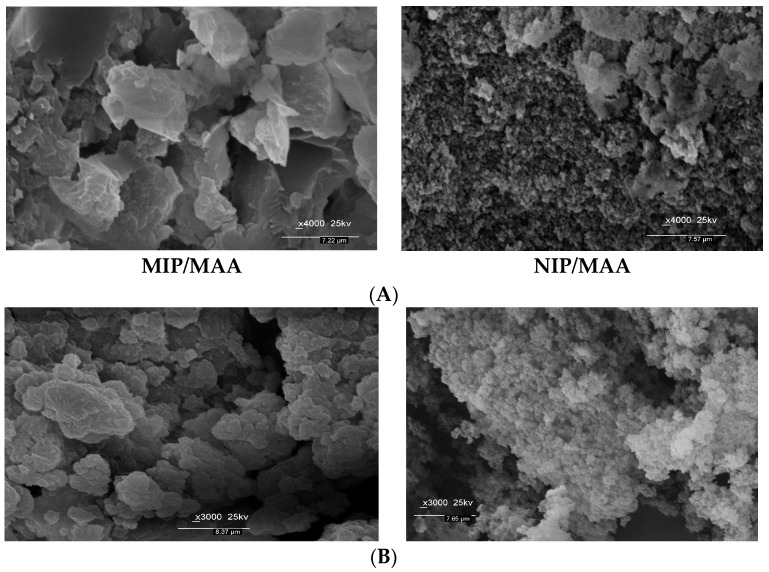
Scanning electon microscope(SEM) images of MIP and NIP using (**A**) MAA and (**B**) AA monomers.

The morphological shapes of the imprinted polymers and also non-imprinted ones were characterized by scanning electron microscopy (SEM). As mentioned in Figure 1, the MIP particles revealed a semi-regular shape with a size distribution of 7–7.5 µm. The NIP particles were found to be more condensed. This indicates that the template could affect the polymer formation. 

**Figure 2:**
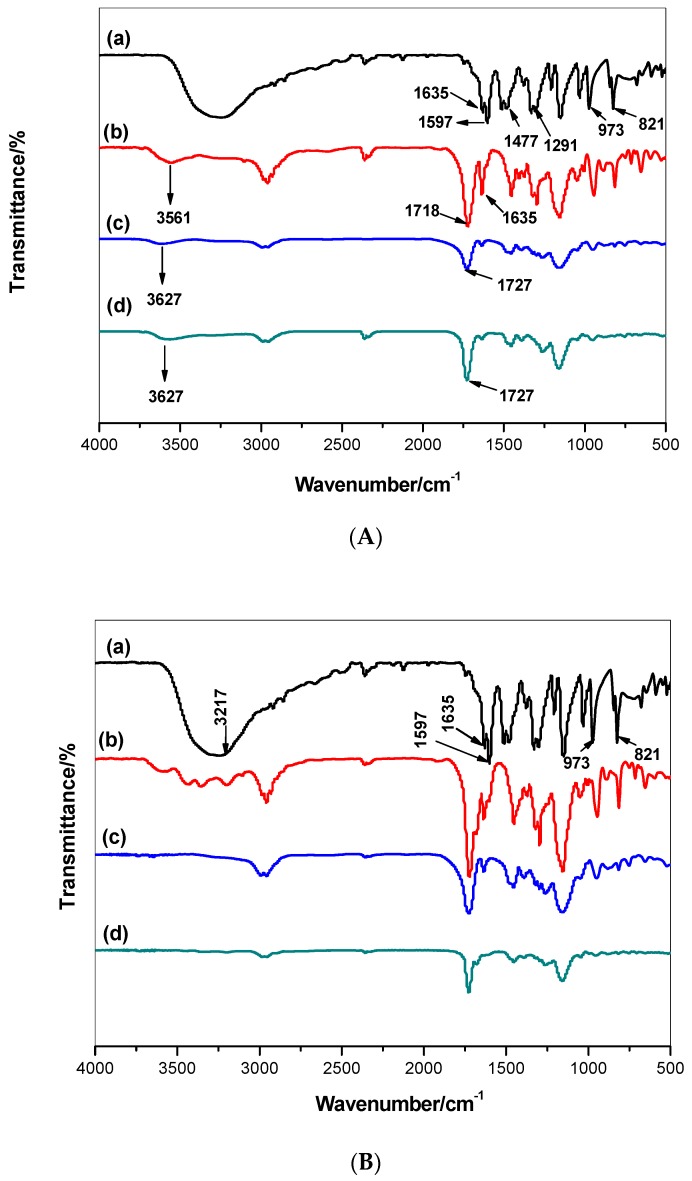
FT-IR spectra of the proposed polymers (**A**) MIP/MAA and (**B**) MIP/AA. (**a**) FT-IR for Orcinol, (**b**) MIP before template removal, (**c**) MIP after template removal, and (**d**) NIP.

The FTIR spectra of MIP particles were collected under room temperature and humidity control after background correction within the ranging 500-4000 cm^−1^. As shown in Figure 2, the imprinting process using MAA and AA monomers through the absence and presence of orcinol on the surface of washed and non-washed polymer particles was confirmed. The FT-IR spectra of orcinol showed a broad band range from 3217 to 3550 cm^−1^, which is assignable to the specific phenolic –OH group, aromatic –C=C– at 1635 and 1597 cm^−1^, -C-O at 973 cm^−1^, and aromatic C–H bending at 821 cm^−1^. All these assignable peaks clearly appeared in MIP/MAA and MIP/AA before template extraction and disappeared in all MIP particles after template removal. The assignable peaks were at approximately 1727 and 1159–1163 cm^−1^, corresponding to –C=O or –C–O stretches, respectively, which are common in all spectra because of the EGDMA cross-linker used. The -O-H stretch outcomes from the methacrylic monomer have a broad peak at 3627 cm^−1^. This peak appeared in both NIP and washed MIP particles. In the non-washed MIP particles, the –O–H stretch peak is shifted to 3561 and another peak appears at 3258 cm^−1^, in agreement with the –O–H stretch in orcinol. From all of the above, the imprinting process of orcinol using MAA and AA as functional monomers was confirmed. 

**Figure 3:**
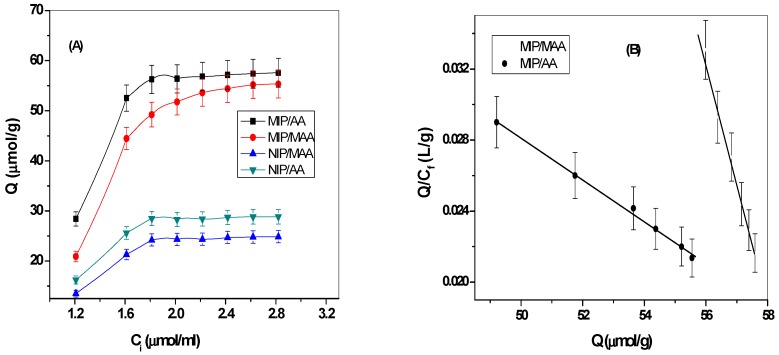
Scatchard analysis for the orcinol-imprinted polymers. Note: *Q* is the amount of orcinol bound to 20 mg of the constructed polymers; T = 25 °C; V = 10 mL; binding time = 20 h.

To prove the non-covalent interaction and rebinding of orcinol to the prepared MIP particles, the binding equilibrium experiment for the two synthesized polymers was carried out. As shown in Figure 3A, the binding amounts for both MIP/MAA and MIP/AA particles increase with increase of orcinol concentrations. The maximum binding capacity (*Q*_max_) for MIP/AA and MIP/MAA was 73.4 and 61.1 µmol/g, respectively. As presented in Figure 3B, the equilibrium dissociation constant (*K*_d_) was also calculated and showed 833.3 and 142.8 µM for MIP/AA and MIP/MAA, respectively. From the data shown above, it was concluded that stable complexes are formed in the imprinting process or orcinol. 

### 3.2. Performance Characteristics of the Sensors

**Figure 4:**
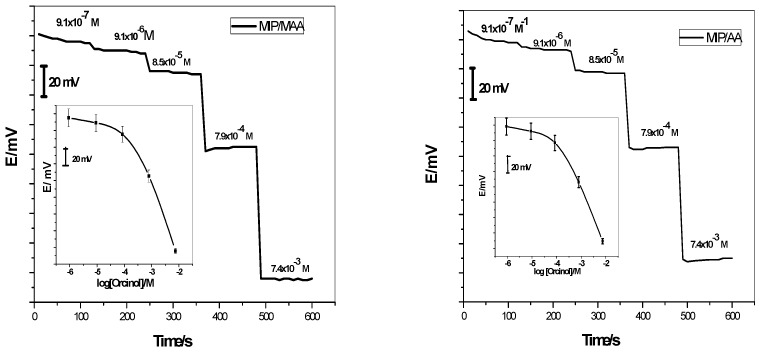
Trace of liquid-contact orcinol-ion-selective electrodes in orcinol concentration. The inset shows the calibration curve of the proposed sensors.

In recent years, well defined theoretical and experimental investigations were reported on the unusual potentiometric response of ISE membranes to lipophilic phenols that may serve as a further basis for sensor development for electrically neutral analytes [15,16,17]. In these investigations, polymeric membranes doped with quaternary ammonium salt revealed super-Nernstian anionic potential responses under near-neutral pH conditions. These strong anionic responses can be attributed to the net movement of H^+^ from the membrane phase to the aqueous phase stimulated by neutral phenols [20,21,22]. According to these findings, we present the application of the non-classical response in orcinol determination. Investigation of the feasibility of using MIPs as sensing elements towards sensitive and selective detection of neutral orcinol was achieved by measuring the responses of different polymeric membrane sensors according to the International Union for Pure Applied Chemistry (IUPAC) guidelines [28]. To guarantee the existence of orcinol in its neutral form, 30 mM PBS (pH = 7) was used as the background solution. As shown in Figure 4, the liquid-contact sensors based on MIP/MAA and MIP/AA revealed super-Nernstian slopes of −92.2 ± 0.7 (n = 5, *r^2^* = 0.9995) and −78.8 ± 1.1 (n = 5, *r^2^* = 0.9995) mV/decade, respectively, over the linear range 5.8 × 10^-4^–10^−2^, 4.4 × 10^−4^–10^−2^ M, and detection limits 1.4 × 10^−4^, 1.1 × 10^−4^ M, respectively. The lower orcinol detection limit (LOD) was calculated from the intersection of extrapolated linear segments of the calibration graph according to IUPAC guidelines and found to be 1.4 × 10^−4^ and 1.1 × 10^−4^ M mol L^−1^ dimethylamine (DMA) with lower quantization limits (LLQ) of 5.8 × 10^−4^ and 4.4 × 10^−4^ mol L^−1^ for MIP/MAA- and MIP/AA-based sensors, respectively. 

As controls, membrane sensors based on NIP/MAA and NIP/AA beads were tested. These sensors showed worse response performances towards orcinol than compared with the response of the MIP/MAA- and MIP/AA-based sensors for all measuring concentrations under the same conditions. They exhibited anionic slopes of −35.2 ± 1.5 (*r^2^* = 0.9986) and −39.4 ± 1.1 (*r^2^* = 0.9978) mV/decade over linear ranges of 1.0 × 10^−2^–6.0 × 10^−3^ M and 1.0 × 10^−2^–4.0 × 10^−3^ M, with detection limits of 1.5 × 10^−3^ and 1.0 × 10^−3^ M, respectively. This confirms that the potential response obtained by these sensors is induced by the specific recognition interactions between the MIP binding sites in the membrane and orcinol.

**Figure 5:**
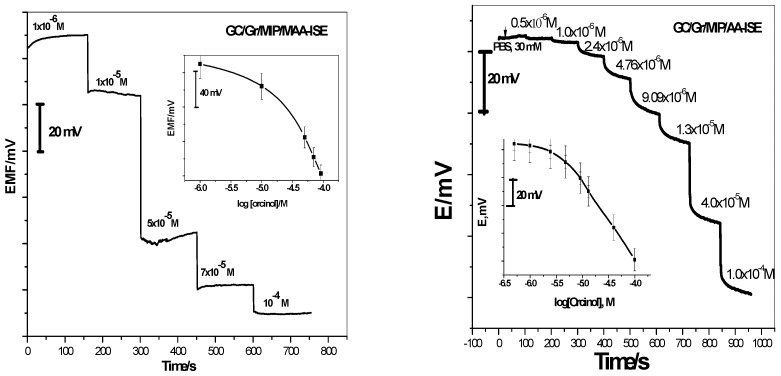
Time trace of GC/Gr/orcinol-ISE in the orcinol concentration range of 10^−6^–10^−4^ M. The inset shows the calibration curve of the proposed sensor.

MIP/MAA and MIP/AA were used as selective materials in the construction of solid-contact sensors (GC/Gr/MIP/MAA-ISE) and (GC/Gr/MIP/AA-ISE), respectively. As shown in Figure 5, the time-dependent potential response curve showed a stable potential response and the response time was about 10 s. The electrodes showed potentiometric responses with slopes −160.3 ± 3.3 (n = 5, *r^2^* = 0.9995) and -55.8 ± 1.1 (n = 5, *r^2^*= 0.9995) mV/decade over a linear range starting from 4.1 × 10^−5^ M and 8.0 × 10^−6^ M with detection limits of 1.7 × 10^−5^ M and 3.3 × 10^−6^ M, respectively. All potentiometric characteristics are shown in Table 1. With all ISEs proposed, the limit of detection, time response, working linear range, and sensitivity slopes were reproducible over a period of at least 12 weeks. 

Within day reproducibility of the proposed ISEs was calculated using 10 µg mL^−1^ internal quality control orcinol sample during short intervals of time within one working day. Significantly small variation (±2.5%) from the final mV readings was noticed. The relative standard deviation (RSD) was found to be 2.1, 2.3, 3.1, and 2.5% for GC/Gr/MIP/MAA-ISE, GC/Gr/MIP/AA-ISE, MIP/MAA, and MP/AA, respectively.

Day-to-day response variations were also tested by measuring the internal quality orcinol sample (10 µg mL^−1^) over 5 consecutive days using different batches of the reagents and was re-calibrated daily. Little variation in the results compared to those obtained for repeatability experiments were obtained. The RSD was found to be 2.8, 2.5, 3.3, and 3.1% for GC/Gr/MIP/MAA-ISE, GC/Gr/MIP/AA-ISE, MIP/MAA, and MP/AA, respectively. These data indicate good response stability of the proposed MIP-based membrane sensors. 

### 3.3. Potential Stability for MIP/MAA/Gr-ISE

**Figure 6:**
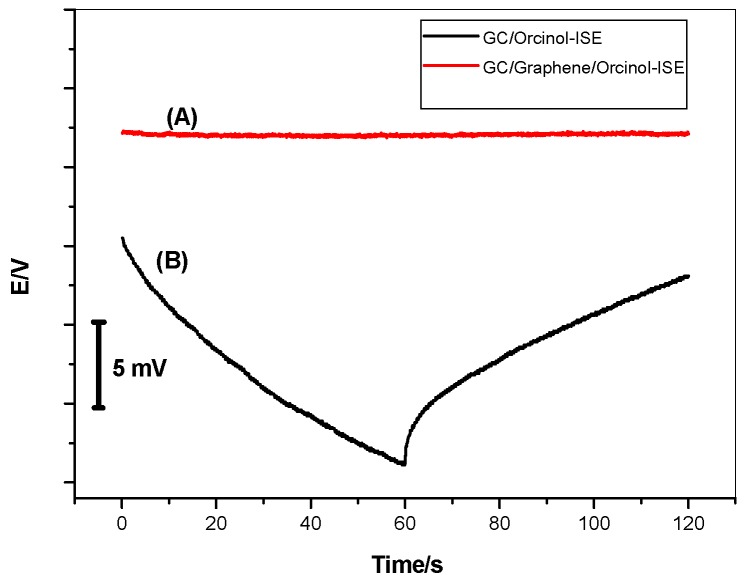
Chronopotentiograms for (A)GC/Gr/MIP/AA-ISE and (B) GC/MIP/AA-ISE under the constant currents of ±1 nA in 1.0 × 10^−3^ M solution.

The short-term stability for the proposed sensors was evaluated using chronopotentiometric measurements. As shown in Figure 6, typical chronopotentiograms for both GC/MIP/AA-ISE and GC/Gr/MIP/AA-ISE were obtained after application of ±1 nA on the working electrodes for 60 s, respectively. The potential drifts of the previously mentioned sensors were 148.5 and 19.1 µV s^−1^, as calculated from the slope (ΔE/Δt) of the curve, respectively. According to the method proposed by Bobacka et al. [29], the capacitance (C) of the ISEs was also calculated and found to be 52.2 ± 0.7 µF and 6.7 ± 0.2 µF, respectively. From these results, the potential stability of the sensor can be dramatically improved by using the graphene solid contact.

### 3.4. Selectivity

Herein using MIPs as sensing elements, the mechanism of selectivity is mainly dependent on stereo-specificity and electrostatic nature of the MIP beads in the membrane sensor. It also depends on the lipophilicity of the tested ions and their capabilities in partitioning between the aqueous solution and lipophilic phase in the sensor membrane. Using the modified separate solution method (MSSM) evaluated by Bakker [30], the selectivity coefficients of the solid contact sensors was evaluated. The potentiometric selectivity coefficient sequences of MIP-based sensors are illustrated in Table 2. The obtained values indicate the preferential interactions of the MIPs toward orcinol over many inorganic anionic species and other phenolic compounds. In general, MIP/AA-based sensors revealed better selectivity than sensors based on MIP/MAA over testing pyrogallol, 2,4-DCP, picric acid, SCN-, I-, 3,4-dihydroxy toluene, 4-methyl catechol, and resorcinol. The selectivity order for the MIP/AA membrane-based sensor over the phenolic compounds is: orcinol > picric acid > 2,4-DCP > phenol ~ 3,4-dihydroxy toluene > pyrogallol > resorcinol > 4-methyl catechol. This selectivity order reflects the acidity and lipophilicity of these phenolic compounds. The general trend is that a phenol derivative with a stronger acidity and higher lipophilicity induces a stronger anionic response. The acid dissociation constants (*pK_a_*) and partition coefficients (1-octanol/water system; log *P*_oct_) for orcinol and other phenolic compounds are indicated in Table 2 [31]. Although the acid dissociation constant and partition coefficient of picric acid and 2,4-DCP are 0.42 and 1.44, and 7.89 and 3.06, respectively [31], the sensors have negligible interference effects on the response towards orcinol. This reflects the specific binding of orcinol by the presented MIP beads as a sensory element.

### 3.5. Optimization of the Flow-Through System

**Figure 7:**
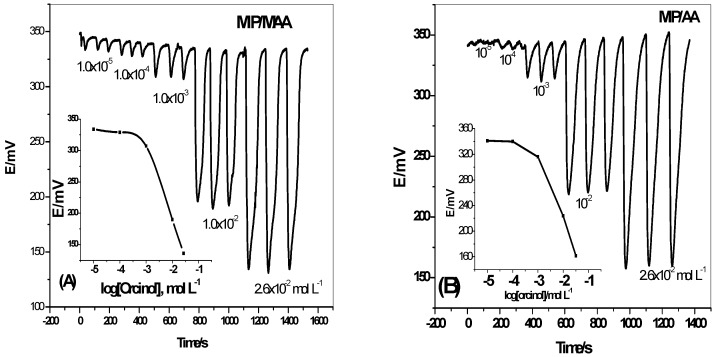
FIA potentiometric signals for (**A**) MIP/MAA and (**B**) MIP/AA based sensors. Conditions: carrier solution, 30 mM PBS pH 7, flow rate 3.5mL/min.

Integration of the proposed sensors in a continuous monitoring system for analyte determination is of a great interest, which can be applied using flow-through analysis. Automation using flow injection technique is a kind of analysis characterized by its versatility, simplicity, ability to accommodate a large number of samples, and also its simplicity. The proposed sensors were used for this purpose and the transient FIA peaks were obtained by injection of 100 μL aqueous solutions of standard orcinol solution into a stream of 30mM PBS solution at pH 7 and flowing at 3.5 mL/min (Figure 7). A linear relationship between orcinol concentrations and FIA signals was obtained from a concentration range of 9.0 × 10^−4^ to 1.0 × 10^−2^ M with an anionic responses −101.3 ± 1.3 and −119.1 ± 0.9 mV/decade for MIP/AA and MIP/MAA, respectively. Table 3 shows the general response characteristics of the sensors under a flow-through mode of operation. 

### 3.6. Analytical Application

To introduce the proposed sensors for real applications, the sensors were finally applied for orcinol determination in soil samples. At first, 100 g soil was soaked into 30 mM PBS at pH 7.0 for 5 h. The mixture was filtered, spiked with different orcinol concentrations, and finally analyzed by the proposed sensor using the standard addition method. The accuracy of the proposed sensor was finally evaluated by determining the recoveries of orcinol in the soil filtrate. Before measurements, a sensor was firstly calibrated by using the linear equation for orcinol. Then, the same batch of sensors could be used directly in the sample analysis. The results are shown in Table 4. It can be seen that the recoveries of soil samples vary from 90.6% to 101.1%, indicating that the proposed MIP-based sensor has promising feasibility for determination of orcinol in complex samples.

## 4. Conclusions

In summary, all-solid-state potentiometric sensors for determination of neutral orcinol have been proposed. The sensors are based on the use of MIP/MAA and MIP/AA as the selective receptors and the graphene film as the solid contact over a GC substrate. This is the first potentiometric sensor for orcinol detection as a neutral species. The proposed MIP-based sensors offered remarkably improved sensitivity for potentiometric detection of neutral orcinol with detection limits of 1.7 × 10^-5^ and 3.3 × 10^−6^ M. Additionally, the proposed sensors showed excellent selectivity, good reproducibility, and satisfactory accuracy for sample analysis.

## Figures and Tables

**Table 1:** Performance characteristics of MIPs based sensors for orcinol evaluation using 30 mM PBS solution at pH 7.

Parameter	Solid-Contact ISEs		Liquid-Contact ISEs			
	GC/Gr/MIP/ MAA-ISE	GC/Gr/MIP/ AA-ISE	MIP/MAA	NIP/MAA	MIP/AA	NIP/AA
**Slope (mV)**	−160 ± 3.3	−55.8 ± 1.1	−92.2 ± 0.7	−35.2 ± 1.5	−78.8 ± 1.3	−39.4 ± 1.1
**Correlation coefficient, (r^2^)**	−0.999	−0.999	−0.999	−0.998	−0.999	−0.997
**Linear range, (M)**	4.1 × 10^−5^– 1.0 × 10^−2^	8.0 × 10^−6^– 1.0 × 10^−2^	5.8 × 10^−4^– 1.0 × 10^−2^	6.0 × 10^−3^– 1.0 × 10^−2^	4.4 × 10^−4^– 10^-2^	4.0 × 10^−3^– 1.0 × 10^−2^
**Detection limit, (M)**	1.7 × 10^−5^	3.3 × 10^−6^	1.4 × 10^-5^	1.5 × 10^−3^	1.1 × 10^−4^	1.0 × 10^−3^
**Response time, (s)**	>10	>10	>10	>10	>10	<10
**Life Span, (week)**	12	12	12	4	12	4
**Standard deviation, σ_v_ (mV)**	1.52	1.1	1	0.8	1	1.2
**Accuracy %**	99.2	98.1	98	99.8	99.4	97.2
**Repeatability, CV_w_ (%)**	0.24	0.3	0.7	0.3	0.55	0.87

**Table 2:** Selectivity coefficients ( *K^pot^_i,j_*) using separate solution methods in 30 mM PBS at pH 7.

Interfering Ion	GC/Gr/MIP/MAA-ISE	GC/Gr/MIP/AA-ISE	*Log P_Oct,w_*	*pKa*_1_
Pyrogallol	−2.03	−2.14	0.97	9.01
I^−^	−0.2	−1.12	-	-
Br^−^	−2.33	−1.81	-	-
NO_2_^−^	−2.17	−1.94	-	-
NO_3_^−^	−1.74	−1.81	-	-
CH_3_COO^−^	−1.94	−1.15	-	-
SCN^−^	−0.53	−2.04	-	-
S_2_O_3_^−2^	−2.88	−2.67	-	-
SO_3_^−2^	−4.33	−3.38	-	-
PO_4_^−3^	−3.44	−2.04	-	-
Phenol	−2.51	−1.79	1.46	9.99
2,4 DCP	−0.3	−1.57	3.06	7.89
Picric acid	1.56	−0.53	1.44	0.42
3,4- dihydroxytolune	−1.60	−1.85	1.37	10.47
4-methyl catechol	−2.10	−2.70	1.37	9.55
Resorcinol	−2.04	−2.31	0.8	9.3

**Table 3:** Response characteristics of orcinol sensors using flow injection analysis in 30 mM PBS at pH 7.

Parameter	MIP/AA	MIP/MAA
Slope, mV/decade	−101.3 ± 1.3	−119.1 ± 0.9
Correlation coefficient, r	−0.987	−0.999
Linear range, M	9.0 × 10^−4^–10^−2^	9.0 × 10^−4^–10^−2^
Detection limit, M	7.7 × 10^−4^	7.2 × 10^−4^
Life span, week	12	12
Optimum flow rate, mL/min	3.5	3.5
Sample frequency, sample/h	41	39

**Table 4:** Determintion of Orcinol content in soil samples using GC/Gr/MIP/AA-ISE.

Added, mM	* Found, mM	Recovery%
0.88	0.89 ± 0.03	101.1
1.60	1.61 ± 0.2	100.6
2.40	2.30 ± 0.1	95.8
3.20	2.9 ± 0.3	90.6
4.00	3.7 ± 0.4	92.5

Note: * average of 6 measurements.
